# Venous congestive ischemic colitis following left hemicolectomy: A rare case of superior rectal vein occlusion

**DOI:** 10.1016/j.radcr.2026.01.026

**Published:** 2026-05-09

**Authors:** Payam Riahi Samani, Ali Hajihashemi, Ramin Jokari

**Affiliations:** a Radiologist, Tehran university of medical sciences, Felestin St., Keshavarz Blvd., Tehran 1416634793, Iran; b Department of Radiology, School of Medicine, Isfahan University of Medical Sciences, Isfahan, 8174673461, Iran

**Keywords:** Ischemic colitis, Venous congestion, Superior rectal vein occlusion, Hemicolectomy complication, Postoperative CT imaging

## Abstract

Ischemic colitis is primarily caused by arterial hypoperfusion, while venous obstruction is rarely documented as the primary cause, particularly as a postoperative complication. This report describes a 66-year-old man who presented with abdominal pain, cramping, and diarrhea 6 days after an elective left hemicolectomy. Contrast-enhanced CT was instrumental in identifying the vascular etiology, revealing an occlusion of the superior rectal vein alongside circumferential wall thickening and submucosal edema in the rectosigmoid colon. Histopathologic analysis confirmed the diagnosis, showing stromal necrosis, small-vessel thrombosis, and acute ischemic changes characteristic of venous congestive ischemic colitis. Following appropriate conservative management, including hydration and bowel rest, the patient achieved full clinical recovery without the need for surgical reintervention.

## Introduction

Ischemic colitis, the most common type of intestinal ischemia, usually arises from temporary decreases in arterial blood flow to the colon. Systemic hypotension, atherosclerosis, thromboembolic episodes, or vasoconstrictive drugs are common causes. Additionally, Nonocclusive Mesenteric Ischemia (NOMI) must be considered, which typically occurs in critically ill patients due to splanchnic vasoconstriction rather than direct vascular blockage. With symptoms like diarrhea, rectal bleeding, and abdominal pain (usually left-sided), the clinical presentation is frequently nonspecific and occasionally includes systemic signs of inflammation [[1], [2], [3], [4], [5], [6], [7]].

On the other hand, venous congestive ischemic colitis is a much less frequent and poorly understood condition. Conditions like portal hypertension, venous thrombosis, or mechanical disruption of venous outflow, especially following colorectal surgery, can result in venous etiologies. Beyond these, other potential triggers include systemic hypercoagulable states, oral contraceptives, and mechanical disruptions like volvulus or extrinsic compression. The diagnosis of venous ischemia can be difficult since its clinical and imaging manifestations can resemble those of cancer or inflammatory bowel disease [5,[8], [9], [10]].

Venous congestive ischemic colitis is rarely documented in the literature and is frequently underdiagnosed, particularly in the postoperative context. Because of its marginal perfusion and vulnerability to both arterial insufficiency and impaired venous drainage, the rectosigmoid colon is particularly vulnerable in these situations [8,11,12].

We describe a 66-year-old man who had a left hemicolectomy and developed venous congestive ischemic colitis in the rectosigmoid area. During surgery, the superior rectal vein was unintentionally blocked, which resulted in poor venous drainage and distal bowel congestion. The condition presented a significant diagnostic challenge because it clinically and radiologically mimicked recurrent neoplasia. This case emphasizes how crucial it is to identify venous causes of colonic ischemia in patients recovering from surgery. Appropriate conservative management can be guided by early radiologic suspicion and histopathologic confirmation, avoiding needless interventions.

## Case report

In order to evaluate a colonic polyp that was first discovered during a routine screening colonoscopy, a 66-year-old man who had no known history of cardiovascular disease, inflammatory bowel disease, or thromboembolic disorders was referred. After the polyp was endoscopically removed, a histopathologic analysis showed high-grade dysplasia which called for surgical intervention. An elective left hemicolectomy was later performed on the patient.

He remained clinically stable during the first postoperative phase. But within days of being released, he started experiencing cramping, diarrhea, and lower abdominal pain, which led to a reassessment and subsequent admission. The symptoms were limited to the left lower quadrant and were not accompanied by fever, hematochezia, or weight loss.

There was no history of such problems, and the patient declined taking antibiotics recently, being exposed to NSAIDs, or traveling. He was not on any long-term medications that would have impacted his bowel loops perfusion or coagulation, and he had no significant medical or surgical history. Neither gastrointestinal malignancies nor ischemic bowel disease ran in the family.

### Clinical presentation

The patient was hemodynamically stable and alert at readmission, but he complained of frequent loose stools and ongoing lower abdominal pain. Upon physical examination, he showed no rebound or guarding and only mild tenderness in the left lower quadrant. There were no symptoms of peritonitis. Digital rectal examination revealed no abnormalities, and bowel sounds were recorded.

Laboratory tests showed significant neutrophilia (83.1%) and leukocytosis with a WBC count of 12,400/µL, which were indicative of an inflammatory process. With an ESR of 18 mm/hr and a CRP of 32 mg/L, the inflammatory markers were elevated. Additionally, the patient had an elevated LDH (845 U/L) and microcytic anemia (MCV: 70.3 fL, MCH: 22.5 pg). With the exception of a lengthy APTT (56 seconds), coagulation tests were largely within normal bounds.

Upon examination, the stool was found to be semi-loose, green, and free of parasites, ova, or fecal leukocytes. An overt infectious colitis was ruled out by these results.

At readmission, the patient was hemodynamically stable and conscious, but he continued to experience lower abdominal pain and loose stools on a regular basis. Upon physical examination, he showed no rebound, guarding, or indications of peritonitis, but rather mild tenderness in the left lower quadrant. Bowel sounds were heard, and the digital rectal examination revealed nothing unusual Table 1, Table 2.

**Table 1:** Laboratory findings upon readmission.

Parameter	Value	Reference range
White blood cell (WBC)	12,400/µL	4,500-11,000/µL
Neutrophils	83.1%	40%-70%
C-reactive protein (CRP)	32 mg/L	< 10 mg/L
Erythrocyte sedimentation rate (ESR)	18 mm/hr	0–15 mm/hr
Lactate dehydrogenase (LDH)	845 U/L	140–280 U/L
Mean corpuscular volume (MCV)	70.3 fL	80–100 fL
Mean corpuscular hemoglobin (MCH)	22.5 pg	27–33 pg
Activated partial thromboplastin time (aPTT)	56 s	30–40 s

**Table 2:** Timeline.

Day	Clinical event
Day 0	Screening colonoscopy performed: a suspicious polyp was identified and resected.
Day 7	Histopathologic examination of the polyp revealed high-risk features → surgical consultation was initiated.
Day 14	The patient underwent elective *left hemicolectomy* without immediate postoperative complications.
Day 20	The patient developed *abdominal pain, diarrhea, and lower abdominal cramping* and presented to the emergency department.
Day 21	Laboratory tests showed *leukocytosis, elevated CRP, prolonged APTT, microcytic anemia, and elevated LDH* → suspicion for inflammation and ischemia.
Day 22	*Contrast-enhanced CT* revealed long segment thickening of the rectosigmoid colon, submucosal edema, pericolic fat stranding, and *superior rectal vein occlusion* → suggestive of *venous congestive ischemic colitis*.
Day 23	*Colonoscopy and rectal biopsy* were performed to rule out recurrence.
Day 24-33	*Detailed specimen processing,* including the application of specialized vascular stains and a thorough review by a gastrointestinal pathologist to confirm the rare diagnosis of small-vessel thrombosis.
Day 34	*Histopathology confirmed acute ischemic colitis*, with vascular thrombosis and mural fibrosis consistent with a *venous etiology*.

Although the patient presented with a prolonged APTT of 56 seconds, a specific underlying coagulation disorder was subsequently ruled out through further testing. The finding was determined to be transient, likely secondary to the acute inflammatory response or the localized ischemic process itself.

**Fig. 1:**
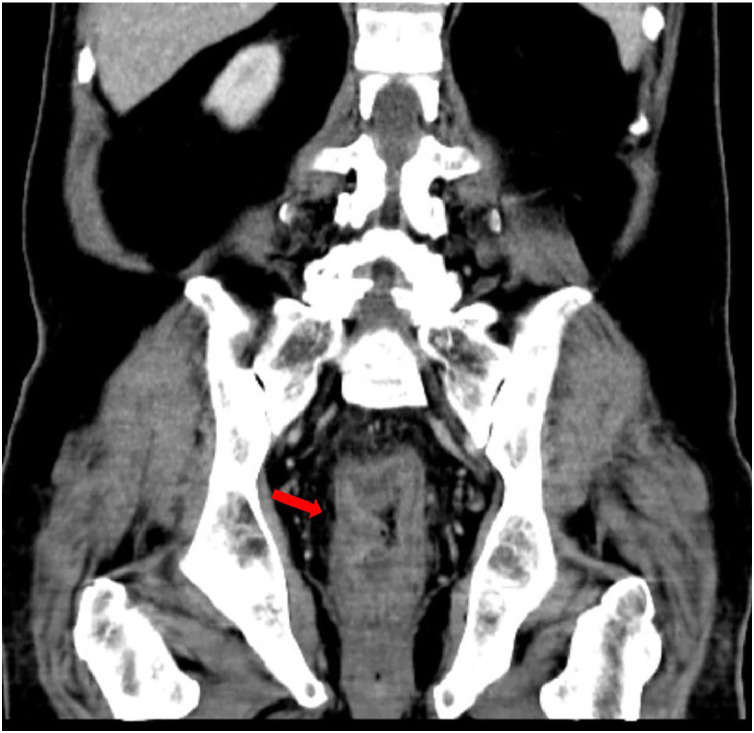
Axial view of abdominal CT scan (A, B), showing long-segment circumferential wall thickening (red arrow), surgical sutures of partial hemicolectomy (blue arrow), and mesenteric engorgement with fat stranding (yellow arrow). Crucially, surgical clips are visible on the superior rectal vein (distal continuation of the inferior mesenteric vein), resulting in its complete occlusion and subsequent venous outflow obstruction (green arrow). Surgical clips located on the inferior mesenteric vein/superior rectal vein complex, resulting in localized venous outflow obstruction.

**Fig. 2:**
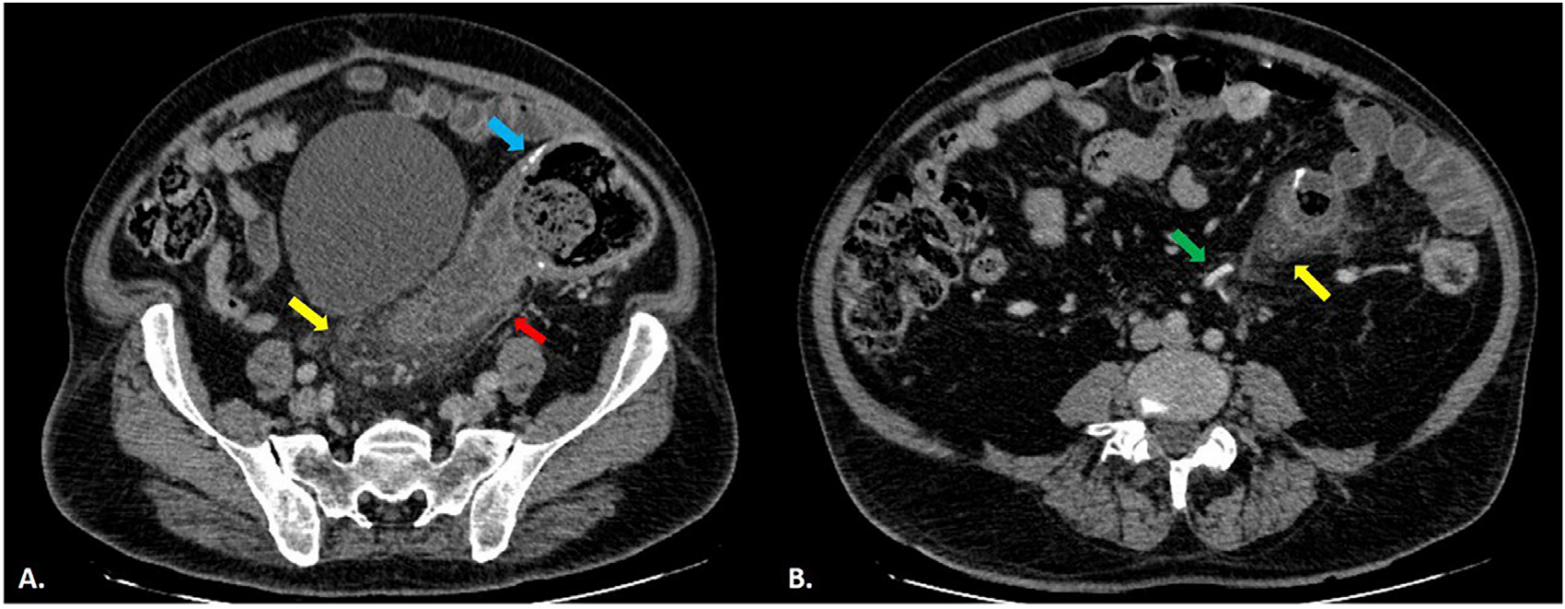
Coronal view of abdominal CT scan, showing long-segment circumferential wall thickening (red arrow).

A contrast-enhanced abdominopelvic CT scan was acquired because of the patient’s ongoing abdominal pain (Fig. 1, Fig. 2). There were visible postoperative changes consistent with a recent left hemicolectomy. Distal to the anastomosis, a long-segment (approximately 15 cm) circumferential and symmetrical wall thickening was observed in the rectosigmoid colon. This thickening involved the entire circumference of the bowel without evidence of rectal sparing, extending from the anastomosis distally. This was linked to moderate pericolic inflammatory changes, decreased mucosal enhancement, and submucosal edema. There were a few normal-appearing and normal-sized pericolic lymph nodes. Crucially, a surgical clamp was found next to a blockage of the superior rectal vein, which indicated venous outflow obstruction and consequent venous congestive colitis. There were no radiologic indications of tumor recurrence found. There were no radiologic indications of tumor recurrence, and notably, there was an absence of ascites or portal venous gas, which helped rule out transmural infarction or perforation at that stage.

Venous ischemic colitis was suspected based on the clinical symptoms and CT results [[13], [14], [15]]. However, a colonoscopy with biopsy was carried out because of worries about a potential tumor recurrence. Endoscopy showed congestion and mucosal edema in the rectosigmoid area.

Classic symptoms of venous congestive ischemic colitis, such as stromal edema, karyorrhexis, necrotic debris, red blood cell extravasation, and small-vessel thrombosis with mural fibrosis, were revealed by histopathologic examination of rectal biopsies.

Stool analysis revealed no signs of pathogens, ruling out infectious etiologies. Based on imaging (no mass or abscess) and histopathology (no dysplasia or malignancy), neoplastic and inflammatory causes were ruled out. These results supported the diagnosis of superior rectal vein occlusion-related venous congestive ischemic colitis.

### Management and outcome

Infectious, neoplastic, and inflammatory etiologies were successfully ruled out based on stool analysis (negative for pathogens), imaging (no mass or abscess), and biopsy (no dysplasia or malignancy).

The patient received intravenous fluid resuscitation for hydration and bowel perfusion support, bowel rest and a gradual reintroduction of a low-residue diet, analgesia with non-NSAID medications and close clinical and laboratory monitoring, including serial abdominal exams, inflammatory markers, and complete blood counts.

Since there was no evidence of infection, no antibiotics were given at first, and no surgical reintervention was necessary. The patient’s symptoms gradually improved over a few days. A follow-up assessment revealed clinical stabilization, with the abdominal pain resolved and bowel habits returning to normal.

### Follow-up and outcomes

Over the course of a week, the patient, who was under constant observation while in the hospital, demonstrated increasing clinical improvement. Diarrhea and abdominal pain went away without the need for surgery. Hemoglobin remained stabilized, white blood cell counts returned to normal, and inflammatory markers showed a downward trend, according to repeated laboratory testing.

The patient was afebrile, hemodynamically stable, able to tolerate oral intake, and passing formed stools free of mucus and blood at the time of discharge. He was discharged with recommendations for; Oral hydration and dietary modification (low-fiber diet transitioning to normal) and Avoidance of NSAIDs, Scheduled outpatient follow-up with gastroenterologist and surgeon. No anticoagulation or surgical revision was considered necessary, and the decision was made to continue conservative management. With full clinical resolution, the overall result was positive.

## Discussion

The most prevalent type of gastrointestinal ischemia, ischemic colitis, is usually caused by arterial insufficiency, especially in older patients who have cardiovascular risk factors. On the other hand, venous congestive ischemic colitis is comparatively uncommon and less commonly reported, especially when it comes to postoperative complications. This case illustrates a rare but significant condition: venous congestive ischemic colitis brought on by occlusion of the superior rectal vein after left hemicolectomy [8]. It is important to note for anatomical clarity that the superior rectal vein serves as the distal continuation of the inferior mesenteric vein; thus, an occlusion or ligation at the level of the surgical clips directly impairs the primary venous drainage route for the rectosigmoid colon.

Being a watershed region with marginal perfusion, the rectosigmoid colon is susceptible to ischemia in conditions involving both low flow and compromised venous drainage [16]. The ischemic injury in this patient was caused by mechanical venous outflow obstruction as a result of surgical clumping or unintentional ligation of the superior rectal vein during colectomy, not arterial hypoperfusion. Mucosal edema, inflammation, and histologic signs of ischemic colitis were caused by the ensuing venous congestion.

Clinically, the patient's symptoms—localized pain, diarrhea, and cramping in the abdomen—were nonspecific and could be traced back to a variety of situations, such as infectious colitis, inflammatory bowel disease, and even tumor recurrence. When postoperative patients present with new gastrointestinal complaints, it is crucial to maintain a broad differential diagnosis and pursue a thorough imaging evaluation.

The diagnosis was made possible by contrast-enhanced CT, which showed no signs of tumoral recurrence but rather segmental thickening of the bowel wall, submucosal edema, and—most importantly—occlusion of the superior rectal vein. A follow-up endoscopic biopsy verified the venous ischemic pattern, revealing small-vessel thrombosis, RBC extravasation, and stromal necrosis, all of which were indicative of congestive venous injury.

This case highlights how crucial it is to differentiate venous ischemic colitis from other postoperative reasons for thickening of the colonic wall. Venous ischemia, particularly when segmental and nontransmural, can frequently be treated conservatively, whereas arterial ischemia may require immediate revascularization or resection. In this instance, full recovery and effective nonoperative management resulted from appropriate recognition.

Isolated venous ischemia of the rectosigmoid colon after colorectal surgery is uncommon but becoming more widely recognized, according to a review of the literature. One case, for example, involved a 73-year-old man who, 11 months after sigmoid cancer was removed, developed symptomatic venous congestive colitis . This was explained by the preservation of arterial flow and venous congestion of the anastomosis region. Another case reported segmental venous congestive ischemic colitis that mimicked inflammatory bowel disease and was caused by inferior mesenteric vein ligation following sigmoidectomy. Furthermore, ischemic changes in the colon have been reported as a result of venous congestion brought on by mesenteric arteriovenous malformations. Conservative treatment was effective in 1 pediatric case of ischemic colitis caused by obstruction of the mesenteric and splenic veins [8,12].

This case supports mounting evidence that venous pathology plays a part in colonic ischemia and emphasizes the diagnostic value of contrast-enhanced CT in differentiating vascular etiologies, especially by detecting venous occlusion, which can direct timely and appropriate treatment without the need for needless surgery.

After learning that the symptoms were not caused by a cancer recurrence, which had been a major concern at first, the patient expressed relief. He was relieved that no additional surgery was required and comforted by the careful clinical observation. After being released, he said his quality of life had significantly improved and he was happy with the treatment and results.

## Informed consent

The patient gave written informed consent for this case report, along with the clinical information and imaging results that go with it, to be published.

## Patient consent

Written consent was obtained from the patient.
